# Roles and regulation of histone acetylation in hepatocellular carcinoma

**DOI:** 10.3389/fgene.2022.982222

**Published:** 2022-08-25

**Authors:** Jin-kun Xia, Xue-qian Qin, Lu Zhang, Shu-jun Liu, Xiao-lei Shi, Hao-zhen Ren

**Affiliations:** ^1^ Department of Hepatobiliary Surgery, Affiliated Drum Tower Hospital of Nanjing University Medical School, Nanjing, China; ^2^ Hepatobiliary Institute Nanjing University, Nanjing, China

**Keywords:** hepatocellula carcinoma, histone (de)acetylation, HDAC inhibition, epigenetic modfication, anticancer

## Abstract

Hepatocellular Carcinoma (HCC) is the most frequent malignant tumor of the liver, but its prognosis is poor. Histone acetylation is an important epigenetic regulatory mode that modulates chromatin structure and transcriptional status to control gene expression in eukaryotic cells. Generally, histone acetylation and deacetylation processes are controlled by the opposing activities of histone acetyltransferases (HATs) and histone deacetylases (HDACs). Dysregulation of histone modification is reported to drive aberrant transcriptional programmes that facilitate liver cancer onset and progression. Emerging studies have demonstrated that several HDAC inhibitors exert tumor-suppressive properties via activation of various cell death molecular pathways in HCC. However, the complexity involved in the epigenetic transcription modifications and non-epigenetic cellular signaling processes limit their potential clinical applications. This review brings an in-depth view of the oncogenic mechanisms reported to be related to aberrant HCC-associated histone acetylation, which might provide new insights into the effective therapeutic strategies to prevent and treat HCC.

## 1 Introduction

Hepatocellular carcinoma (HCC), the most common form of primary liver cancer and accounts for ∼90% of cases, is a severe neoplastic disease and the average 5-years survival for HCC patients is less than 15% (Erstad et al., 2019; Llovet et al., 2021). Currently, Hepatitis B virus (HBV) and hepatitis C virus (HCV) are considered as the most important pathogenic factors for HCC, but their significance will possibly decline in the coming years (Sagnelli et al., 2020). Unfortunately, the incidence rates of metabolic risk factors for HCC, including metabolic syndrome, obesity, type II diabetes and non-alcoholic fatty liver disease (NAFLD) are increasing and may jointly become the leading cause of HCC worldwide (Mcglynn et al., 2020). Despite great advances in prevention, diagnosis and therapeutic strategies, most are diagnosed at advanced stages where therapeutic options are limited, and the overall survival of patients with HCC has not improved significantly in recent decades (European Association for the Study of The et al., 2012). Sorafenib has long been the treatment strategy for advanced HCC patients; however, sorafenib resistance is considered a serious obstacle that must be overcome for HCC therapy (Zhang et al., 2012). Therefore, it is necessary to comprehend the cellular mechanisms of hepatocarcinogenesis in order to develop new and effective therapeutic targets.

Over the past decades, the development of epigenetics (e.g., microRNA, DNA methylation, and histone modification) has provided a fresh view to uncover the mechanisms of liver carcinogenesis (Bayo et al., 2019; Ganai, 2020b; Liu et al., 2021). Epigenetic phenomena refer to heritable adaptive reversible changes in gene expression that are not induced by changes in the DNA sequence (Cavalli and Heard, 2019). In eukaryotic cells, histone modifications, such as acetylation, phosphorylation, methylation, SUMOylation, and ubiquitination are a major source of molecular functional diversity, and their aberrant regulation is a common feature of many diseases (Bhat et al., 2021). Histone acetylation represent a prevalent event in epigenetic regulation and manipulates oncogenes and tumor suppressor genes during cancer progression (Lawrence et al., 2016). Generally, histone acetylation and deacetylation processes are catalyzed by the opposing activities of histone acetyltransferases (HATs) and histone deacetylases (HDACs) (Verza et al., 2020). In human cells, HATs mainly include three subfamilies: the MYST family, the GNAT family, and the p300/CBP family, and all subfamilies include transcription factor and steroid receptor co-activators with catalytic activity (Gajer et al., 2015). According to the specialized functions of HDACs, they are divided into 4 major classes of 18 members, namely class I (HDAC1, 2, 3, 8), class II (IIa 4, 5, 7, 9, and IIb 6,10), class III [Sirtuin1-7 (SIRT1-7)], and class IV (HDAC11) (Mcclure et al., 2018). Group I HDACs (Class I, II, and VI) are zinc-dependent amidohydrolases. The majority of class I HDACs exist in the nucleus, except for HDAC3 and HDAC8, which can shuttle between the nucleus and cytoplasm (Ganai, 2019). The distribution of Class I HDACs show highly specific tissue expression. Class II HDACs are mostly located in both the nucleus and cytoplasm and require Class I HDACs to obtain catalytic activity (Martin et al., 2007). The second group of mammalian HDACs, Sirtuins, are named for their homology to the yeast silent information regulator 2 (Sir2) gene. Sirtuins are structurally and functionally distinct from Group I HDACs in that their deacetylase activity is NAD + dependent (Bheda et al., 2016). HATs are involved in histone acetylation by the transfer of acetyl groups from acetyl-CoA to lysine residues located on the histones, leading to an open state of chromatin and allowing access of transcription factors and promoting gene transcription. Conversely, HDACs erase the acetyl groups from the lysine residues located on N-terminal ends of histone proteins and recover the positive charge of lysine, resulting in a closed state of chromatin and silencing gene expression (Gray and Dangond, 2006; Falkenberg and Johnstone, 2014; Ganai, 2020c). Histone acetylation has been described to be capable of post-transcriptionally modulating various biochemical pathways that are essential for tumorigenesis (Biswas and Rao, 2017). Because HATs/HDACs can reversibly control the modifications (Tomaselli et al., 2020), it is appealing to develop epigenetic drugs as one of many tools in the fight against liver cancer.

HCC progression is a complex process with dysregulated cellular and molecular events driven by aberrant genetic and epigenetic activities. In particular, the pathogenesis of Hepatitis B virus X protein (HBx)/hepatitis C virus/nonalcoholic steatohepatitis-mediated HCC is tightly related to HATs/HDACs activities (Tsukiyama-Kohara, 2012; Liu et al., 2015; De Conti et al., 2017). Recently, quantitative acetylome analysis and lysine acetylome study revealed that abnormal histone modifications may predict prognosis in HCC patients (Zhao et al., 2020; Chai et al., 2021). Furthermore, several liver-targeting HDAC inhibitors potently suppress HCC growth and animal and preclinical studies with HDAC inhibitors suggest survival benefits (Yeo et al., 2012; Afaloniati et al., 2020; Tapadar et al., 2020). However, the role of acetylated proteins and the precise mechanism of individual HDACs in HCC progression is still not clear. In the present review, we provide an explicit summary of the roles and the underlying regulatory mechanisms of histone acetylation modification in HCC, which will provide us with new strategies for the treatment of HCC.

### 2 Regulatory mechanism underlying the development of hepatocellular carcinoma

#### 2.1 Histone acetylation is implicated in hepatocellular carcinoma metastasis and angiogenesis

Cancer metastasis is the primary obstacle to successful treatment of HCC. Epithelial-mesenchymal transition (EMT), which is characterized by the loss of epithelial cell markers epithelial-cadherin (E-cadherin), as well as the increased expression of the mesenchymal proteins such as N-cadherin, Vimentin, α-smooth muscle actin (α-SMA), and the EMT-transcription factors Snail, Slug, Twist, ZEB (Wang et al., 2018), is an essential process in invasion and metastasis of cancer cell (Dong et al., 2019). Recent evidence has suggested that aberrant acetylated activity of EMT-related genes and EMT upstream genes were tightly associated with tumorigenicity and HCC metastasis (Han et al., 2018). To drive HCC metastasis from primary tumors, HDACs-mediated histone acetylation restrain E-cadherin expression or prompt mesenchymal proteins transcription, thereby facilitating migration and invasion in HCC (Han et al., 2019; Hu et al., 2019). On the contrary, EMT process and cell migration in HCC was suppressed by overexpression of the non-acetylation Vimentin (Guo et al., 2018). β-catenin pathway is one of the critical regulatory pathways in EMT process and cancer metastasis, and the acetylated status of β-catenin or the upstream signal protein kinase B (PKB) might mediate the canonical Wnt pathway in HCC (Chen et al., 2013; Yuan et al., 2020; Han et al., 2021). Interestingly, as the substrate for acetylation reactions, acetyl-CoA plays an important role in epigenetic modifications due to its dynamic association with histone acetylation; and acyl-CoA thioesterase 12 have been reported to epigenetically inducing TWIST2 expression and the promotion of EMT in HCC (Lu et al., 2019). Therefore, biological products that interact with HDAC to correct aberrant acetylated activities provide an attractive approach for cancer therapy (Huo et al., 2021; Zhang et al., 2021). Panobinostat, a new hydroxamic acid-derived histone deacetylase inhibitor (HDACI) has shown promising anticancer effects by inhibiting HCC growth and metastasis recently (Song et al., 2013). However, opposite results highlighted that HDACI promote the expression of Snail and induce EMT in hepatoma cells, thus limiting the clinical outcome of HDACI-based therapies in HCC (Xu et al., 2018; Xiao et al., 2020).

Angiogenesis has a key role in the formation of a new vascular network and HCC is largely dependent on angiogenesis for its energy supply during metastasis process (Morse et al., 2019). Angiogenic gene, such as vasohibin 2 and integrin αV subunit gene, were transcriptionally activated by histone modification and promotes angiogenesis in HCC (Xue et al., 2013; Cai et al., 2018; Cai et al., 2019). Angiogenesis is driven by hypoxic microenvironment, and the cellular response to hypoxia is triggered by the transcription factor hypoxia-inducible factors (HIFs) (Pugh and Ratcliffe, 2003). HIFs play a critical role in the adaptation of cancer cells to hypoxic conditions by activating the transcription of several pro-oncogenic genes (Albadari et al., 2019). Increasing evidence showed that the stability and activity of HIF-1α and HIF-2α were precisely regulated by acetylation modification, thereby contributing to the subsequent EMT process and HCC metastasis (Yoo et al., 2008; Liu et al., 2013; Sun et al., 2017; Cao et al., 2020). In contrast, HDACI destabilizes HIF-1α and diminishes its transcriptional activity during hypoxic microenvironment (Lee et al., 2016). Although the antiangiogenic activity of HDACI has been determined to be associated with decreased expression of proangiogenic genes, the specific effect of individual HDAC enzymes on HCC angiogenesis is still controversial (Lv et al., 2016). Therefore, more selective novel HDACIs might improve the prognosis of patients to a greater extent.

**FIGURE 1:**
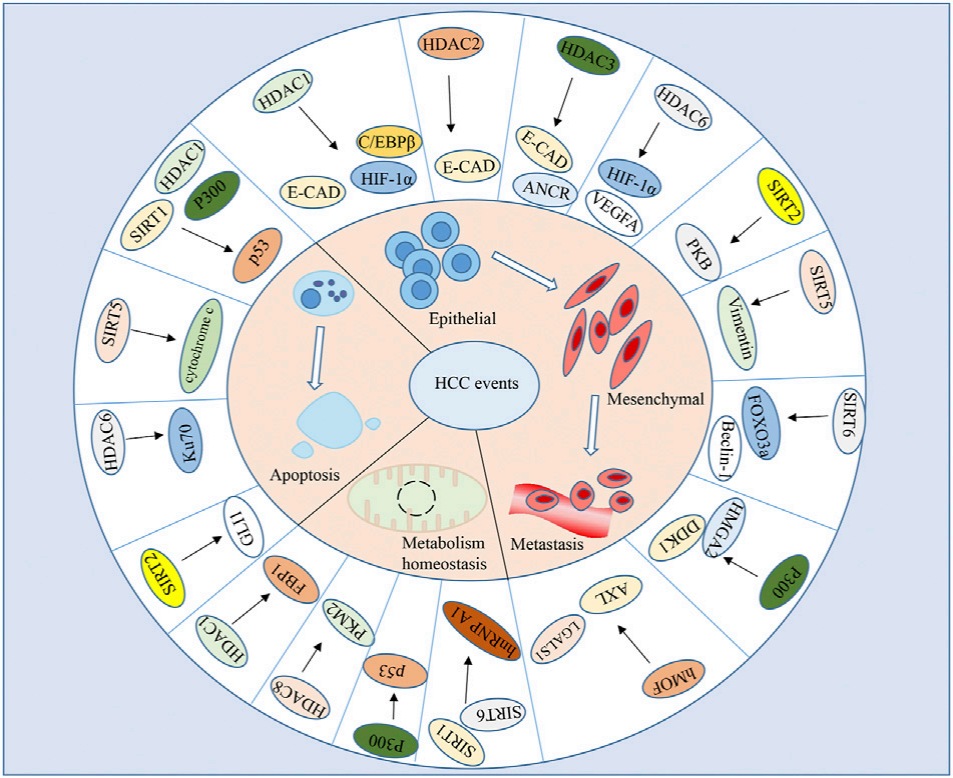
Target genes of HATs/HDACs in HCC. HATs/HDACs mediated-histone modifications affect key protein function that govern a wide array of biological processes in HCC metastasis, apoptosis, and metabolic homeostasis.

In addition, histone acetyltransferase p300 and hMOF are involved in inducing HCC migration and vascular invasion by mediating the acetylation of some oncogenes and enhancing their transcriptions (Niu et al., 2020; Pote et al., 2020; Liang et al., 2021). Epigenetic activation of the microRNAs by histone acetylation also contributes to EMT process and HCC metastasis (Zhang et al., 2013; Wen et al., 2020). Hence, targeting aberrant acetylation present a promising new class of compounds for anticancer therapy. Given the diverse molecular targets and downstream cellular pathways of HDACs (Figure 1), understanding of the context-dependent roles of individual HDACs on HCC metastasis might give us an advantage to treat cancers by exploiting this field in a specifically targeted manner.

### 2.2 Histone acetylation is implicated in hepatocellular carcinoma metabolism

Special metabolic change, including the Warburg effect, unsaturated fatty acid biosynthesis, and so on, induces molecular changes in cancer cell, thereby allowing it to grow and proliferate in a nutrient-poor environment (Pavlova and Thompson, 2016). Cancer cells metabolize glucose to lactic acid to produce ATP and generate metabolic intermediates for the synthesis of lipids, nucleic acids, and proteins during aerobic glycolysis (Warburg effect). As such, it represents a potential therapeutic strategy for cancer. There is an amount of information indicated that histone acetylation was an important factor in cancer metabolism. More than 1000 acetylation sites in proteins were identified in human liver tissues, and metabolic enzymes accounted for a large amount (Zhao et al., 2010). More importantly, acetylation of significant enzymes in the metabolic glycolysis pathway is considered a mainly regulatory mechanism for promoting their enzymatic activities and liver cancer cell metabolism (Figure 1)(Table 1) (Hu et al., 2017; Zhang R. et al., 2020; Gao et al., 2021). The acetylation status of pyruvate kinase M2 isoform (PKM2), a key enzyme for glycolysis, affects the metabolic phenotype of HCC cells. HDAC8 reprograms the glucose metabolism of HCC cells by regulating K62 acetylation of PKM2 protein, and TSP50 promotes the Warburg effect by increasing PKM2 K433 acetylation level (Zhang R. et al., 2020; Gao et al., 2021). Sirtuin-mediated deacetylation of hnRNP A1 also suppresses glycolysis and growth in HCC by PKM2 pathway (Yang et al., 2019). Some HDACI is protective against HCC via correcting aberrant acetylated activity of fructose-1,6-bisphosphatase (FBP1) gene, thus, suppressing glucose metabolism and HCC cell growth *in vitro* and tumor growth in mice (Yang et al., 2017). Particularly, the tumor suppressor gene p53 was shown to revert the Warburg effect and negatively influence the oncogenic metabolic adaption of cancer cells (Gomes et al., 2018). p53 was the first non-histone protein shown to be regulated by histone acetyltransferases and histone deacetylases, and this type of modification is essential for p53 activity in HCC. The acetylated p53 is responsible for the deregulation of glycogen metabolism and represents a promising therapeutic target for the clinical management of HCC (Chen et al., 2019; Di Leo et al., 2019). Of note, the glycolytic product lactate also plays a crucial role in regulating gene transcription by inhibiting the HDAC enzymes, promoting hyperacetylation in nucleosomes and active transcriptional state (Liu and Zhang, 2018). The HAT activity of p300/CBP is often aberrantly controlled in human disease, and targeting p300/CBP has been shown to produce antitumor effects *in vitro* against several hematological malignancies, prostate and colorectal cancers (Du et al., 2017; Lasko et al., 2017). Increased expression of p300 has also been reported to correlate with poor survival and aggressive phenotypes in HCC, and p300 inhibitor attenuates HCC through epigenetic regulation of glycolytic function and nucleotide synthesis (Cai et al., 2021).

**TABLE 1:** Key proteins modified by acetylation during HCC metabolism.

Acetylated Proteins	upstream Regulator	metabolic Process	references
PKM2	HDAC8	Glucose metabolism	(Zhang et al., 2020c)
	SIRT2	Warburg effect	(Gao et al., 2021)
	SIRT1 and SIRT6	Glycolysis	(Yang et al., 2019)
p53	P300	lycogen metabolism	(Chen et al., 2019)
		Glycolytic rewiring	(Di Leo et al., 2019)
FASN	ACAT1	Lipid metabolism	(Gu et al., 2020)

Increasing evidence suggests that hyperactive lipogenesis contribute to the establishment and maintenance of the tumorigenic state. Fatty acid synthase (FASN) is a key enzyme for the synthesis of long-chain fatty acids from malonyl-CoA, and FASN overexpression has been identified in many cancer types (Kuhajda et al., 1994). Stabilization of FASN by ACAT1-mediated GNPAT acetylation promotes lipid metabolism and HCC progression (Gu et al., 2020). Acetyl-CoA is an important metabolic intermediate that act the substrate of histone acetyltransferases regulating gene expression. It’s reported that liver mitochondrial fatty acid-derived acetyl-CoA would, like glucose-derived acetyl-CoA, be used for lipid anabolism and fuel nuclear acetylation events in citrin-deficient liver (Mention et al., 2021). Similarly, eicosapentaenoic acid (EPA), a fatty acid with anti-cancer properties, inhibited HDAC1 and DNMT expression and activity, thus promoting tumor suppressor gene expression in HCC (Ceccarelli et al., 2020).

Metabolic reprogramming plays an important role in supporting liver tumor growth. However, little is known about the histone modifications that cause HCC metabolic alterations; and whether metabolic intermediate influence the HCC progression by epigenetic manner. Those information will be helpful for better understanding the mechanisms by which oncogenic metabolites regulate the malignant phenotypes of cancer.

### 2.3 Histone acetylation is implicated in hepatocellular carcinoma apoptosis

Apoptosis is a precise process of programmed cell death that is crucial for progression of certain cancers including HCC. Accumulating evidence indicates that apoptotic genes can be regulated by epigenetic mechanisms (Zhou et al., 2019). Some HDAC inhibitors such as panobinostat (Choi et al., 2021), SAHA analogues (Srinivas et al., 2016), and traditional Chinese medicine galangin (Li et al., 2016) have been recently reported to regulate apoptosis in HCC through controlling the expression of pro- and anti-apoptotic genes (Buurman et al., 2016; Li and Seto, 2016). In general, administrations of HDACI can either directly prompt apoptosis through the extrinsic (death receptor)/intrinsic (mitochondria) pathway, or induce the susceptibility of tumor cells to apoptosis (Li and Seto, 2016). SIRT5 and SIRT6 were considered as the crucial lysine deacetylases that promotes HCC progression by regulating mitochondrial apoptosis (Tao et al., 2017; Zhang et al., 2019). HDAC inhibitor droxinostat could induce apoptosis in HCC cells via activation of the mitochondrial apoptotic pathway (Liu J. et al., 2016). Histone acetyltransferase PCAF also accelerates apoptosis by repressing pro-apoptotic gene BCL2-Associated X (Bax) axis or acetylating histone H4 and inactivating AKT signaling in HCC (Zheng et al., 2013; Gai et al., 2015).

One of the several biological functions of p53 is the ability to prompt apoptotic cell suicide. It’s reported that intracellular hepatitis B e antigen (HBeAg) and its precore precursors could inhibit the acetylation and translocation of p53 from cytosol to the nucleus, resulting in degradation of p53 and suppression of p53-dependent apoptosis (Liu D. et al., 2016). Long non-coding RNA (lncRNA) LOC100294145 also impedes p53 acetylation by interacting with HDAC1 and p300 to prevent HDAC1 degradation and attenuate p300 activity, leading to abrogation of p53 activity and subsequent cell proliferation and apoptosis resistance (Zhang L. Z. et al., 2020). In addition, the p53 deacetylase, SIRT1, was phosphorylated and inactivated by AMPK, resulting in p53 acetylation and apoptosis of HCC cells (Lee et al., 2012). Intriguingly, histone acetylation may regulate HCC apoptotic processes not only via p53-dependent way, but also through p53-independent pathways (Figure 1) (Lou et al., 2015; Liu D. et al., 2016; Lin et al., 2019). Treatment of pan-deacetylase inhibitor panobinostat or inducing p53 protein acetylation provide a novel therapeutic strategy for HCC by inducing apoptosis and inhibiting hepatoma cell growth (Zhu et al., 2009; Park et al., 2012; Song et al., 2013; De Matteis et al., 2018; Lim et al., 2020).

### 2.4 Histone acetylation is implicated in hepatocellular carcinoma immune homeostasis

Insufficient T cell infiltration in HCC limits the effectiveness of immune-checkpoint blockade (ICB) for a subset of patients. Epigenetic therapy provides further opportunities to activate cancer-associated transcriptional programs through immune regulation. It has been demonstrated that a selective HDAC8 inhibitor potentiates antitumor immunity and efficacy of immune checkpoint blockade in HCC (Yang et al., 2021). Similarly, disruption of SIRT7 increases the efficacy of checkpoint inhibitor via MEF2D regulation of programmed cell death 1 ligand 1 in HCC cells (Xiang et al., 2020). The information regarding acetylation modulation of immune in HCC is increasing, but the mechanism of selective epigenetic inhibition counteracts the immune-excluded phenotype is still unclear. Understanding the epigenomes of HCC may improve the response rate of the combination of ICB with HDACI.

### 2.5 Histone acetylation is implicated in cancer signaling pathway of hepatocellular carcinoma

The alterations of intracellular and extracellular cancer-associated signaling pathway have profound effects on gene transcription, cellular differentiation, and tumor microenvironment, all of which participate in the establishment and maintenance of the tumorigenic state. It has been confirmed that many cancer signaling pathways are linked with the modifications of acetylation (Table 2). An active area of research is to understand HATs/HDACs mediated-histone modifications affect key protein function and how they do so. In many cases, HDACs reverse chromatin acetylation and alter transcription of oncogenes and tumor suppressor genes by removing acetyl groups. HDACs also deacetylate nonhistone cellular substrates that govern a wide array of biological processes in liver cancer initiation and progression. HATs and HDACs activity antagonize each other to balance intracellular acetylation status. The cellular levels and biological activities of these enzymes provide a direct link between epigenetic modifications and the control of cancer signaling, transcription, and cell growth. Furthermore, acetylation of histone variant H2A.Z is also implicated in the transcriptional misregulation in cancer signaling pathway in HCC (Yuan et al., 2021). Aberrant regulation by acetylation on these signaling pathways and biological processes resulted in carcinogenesis and progression of HCC. Therefore, acetylation may function as a promising target of anti-HCC treatment.

**TABLE 2:** The target cancer signaling pathways by acetylation modifications in HCC.

HATs/HDACs	Target Signaling Pathways	Cellular Function	References
P300	TGF-β1 signaling	Cell proliferation	Guo et al. (2021)
MOF	Estrogen receptor α signaling pathway	Cell growth, migration, and invasion	Wei et al. (2021)
N-α-acetyltransferase 20 (Naa20)	AMPK-mTOR signaling pathway	Cell proliferation, autophagy	Jung et al. (2020)
-	PTEN signaling	Cell proliferation and angiogenesis	Zhang et al. (2020a)
CBP and SIRT1	PTEN signaling and pro-apoptotic protein caspase-3	Cell proliferation, migration, invasion, and apoptosis	Xue et al. (2020)
-	p38 MAPK signaling	Cell stemness and metastasis	Luk et al. (2020)
HDAC3	TRAF6/c-Myc signaling	Cell proliferation	Wu et al. (2020)
HDAC1	PTEN/Akt signaling	Cell proliferation, migration and invasion	Tian et al. (2017)
PCAF	STAT3 signaling	Cell proliferation	Zheng et al. (2016b)
HDAC11	AMPK Signaling	Cell stemness	Bi et al. (2021)
P300/SIRT1	YAP signaling	Cell proliferation, apoptosis	Wang et al. (2015)

## 3 Anticancer effect of histone deacetylase inhibitors in hepatocellular carcinoma

The possibility to modulate epigenetic alterations of tumor cells by HDACIs provide new treatment options for HCC that exhibit an inherent resistance to cytostatic agents (Table 3). HDACs reversibly modify the acetylated histones and nonhistones, and cause widespread alterations in genes expression without a change in DNA sequence. The disrupted acetylation homeostasis in cells might contribute to tumorigenesis, and HDACIs can counteract the abnormal acetylation status of proteins existed in liver cancer cells, and can reactivate many tumor suppressors (Lai et al., 2006). Moreover, HDAC inhibitors induced considerable cellular damage in HCC-derived cells, but did not impair cellular integrity of primary human hepatocytes (Armeanu et al., 2005). However, mechanisms of anticancer effects of HDAC inhibitors are not uniform, which may depend on the cancer type, HDAC inhibitors, doses, etc. In addition to designing inhibitors against the aberrant activity of HDAC, targeting other key molecules that regulate acetylation has also been shown to exert significant effects in anti-HCC therapy, although data are limited. For example, B029-2 (a novel p300 inhibitor) disrupts the metabolic reprogramming of HCC cells by reducing H3K18Ac and H3K27Ac levels at the promoter regions of amino acid metabolism and nucleotide synthesis enzyme genes, and thus is a potential drug for the treatment of HCC (Cai et al., 2021). Bromodomains are epigenetic "readers” of histone acetylation and bromodomain inhibitors also have exhibited promising therapeutic potential for liver cancer treatment (Cheng et al., 2021).

**TABLE 3:** Anti-cancer effects of HDAC inhibitors in HCC.

HDAC Inhibitor	Specificity	Effects in HCC	References
Panobinostat	Classes I, II, IV	inhibit HCC growth and metastasis	Song et al. (2013)
		decreased expression of an anti-apoptotic protein	(Choi et al., 2021)
		elicits effective responses to sorafenib	Lachenmayer et al. (2012)
Vorinostat (SAHA)	Classes I, II, IV	induce EMT	(Xu et al., 2018; Xiao et al., 2020)
		sensitize HCC cells to sorafenib	Yuan et al. (2014)
		sensitize HCC cells to 5-FU	Wang et al. (2021)
SAHA analogues	Classes I, II, IV	inhibits cell proliferation and induces apoptosis	Srinivas et al. (2016)
sodium butyrate	Classes I, II	induce EMT	(Xu et al., 2018; Xiao et al., 2020)
		suppresses HCC growth	Yang et al. (2017)
valproate (VPA)	Classes I, II	inhibits cell proliferation and induces apoptosis	Armeanu et al. (2005)
eicosapentaenoic acid	HDAC1	promotes tumor suppressor gene expression	Ceccarelli et al. (2020)
Santacruzamate A	HDAC2	increasing the sensitivity of radiotherapy	Jin et al. (2021)
Droxinostat	HDAC3	induces apoptosis	Liu et al. (2016b)
PCI-34051	HDAC8	elicits effective responses to ICB	Yang et al. (2021)
Rhamnetin	SIRT1	enhances the antitumor effect of sorafenib	(Li et al., 2021)

After the FDA approval of HDAC inhibitors such as vorinostat and romidepsin as anticancer agents, many novel epigenetic drugs have been investigated to reverse immune resistance and synergize with ICB treatment (Zheng H. et al., 2016). It has been confirmed that disruption of SIRT7 expression or administration of a selective HDAC8 inhibitor enhances antitumor immunity and efficacy of ICB in HCC (Xiang et al., 2020; Yang et al., 2021). In addition to the effects on the tumor cell growth, HDACI promotes the expression of MHC class I-related chain molecules A and B (MICA and MICB), resulting in an increased susceptibility of HCC cells to immune therapy (Yang et al., 2015). However, nonselective HDAC inhibition have also shown immunosuppressive effects in cutaneous T-cell lymphoma patients by reducing the activation and cytokine production of natural killer cells and dendritic cells (Kelly-Sell et al., 2012), or increasing the production and immunosuppressive functions of regulatory T (Treg) cells and myeloid-derived suppressor cells (MDSCs) (Tao et al., 2007; Rosborough et al., 2012). Given the diverse outcomes of HDAC inhibition in immunoregulation, delineating isozyme-specific HDAC control of HCC tumor microenvironment may provide insights into rational design of combination immunotherapies.

In recent years, drug combination is an effective strategy to reduce cell toxicity and improve the efficacy of therapy. Epigenetic combination therapy that comprise HDACI and demethylating agents was found to exert significant antitumor effects in HCC (Venturelli et al., 2007). A portion of HCC patients can benefit from treatments with sorafenib, adriamycin, 5-fluorouracil and platinum drugs; however, most of them eventually develop drug resistance, which partly owing to overexpression of HDACs (Ceballos et al., 2018). The combination of HDAC inhibitor such as vorinostat (SAHA) and rhamnetin (an inhibitor of SIRT1) with the antineoplastic drugs could overcome the drug resistance (especially sorafenib resistance) in HCC and notably augmented the anticancer responses (Lachenmayer et al., 2012; Yuan et al., 2014; Li et al., 2021; Wang et al., 2021). Furthermore, inhibition of HDAC2 or HDAC4 expression increases the sensitivity of liver cancer radiotherapy (Tsai et al., 2018; Jin et al., 2021). However, many epigenetic drugs of small chemical compounds are cytotoxic, and epigenetic diets are emerging as relatively safe supplementations (Lewis and Tollefsbol, 2017). Pterostilbene, a small compound isolated from plants, could serve as an novel epigenetic drug by suppressing HDAC1 activity (Qian et al., 2017; Qian et al., 2018), thus opens up new avenue for the prevention and treatment of epigenetic disorders in HCC. Intriguingly, plant flavonoid luteolin also exert therapeutic impact by restoring ethanol-depleted SIRT1 activity in pre-neoplastic liver lesion mouse model (Ganai et al., 2021). These results suggest that epigenetic diets might correct aberrant HDACs abilities to maintain acetylation homeostasis in HCC.

## 4 Discussion

**FIGURE 2:**
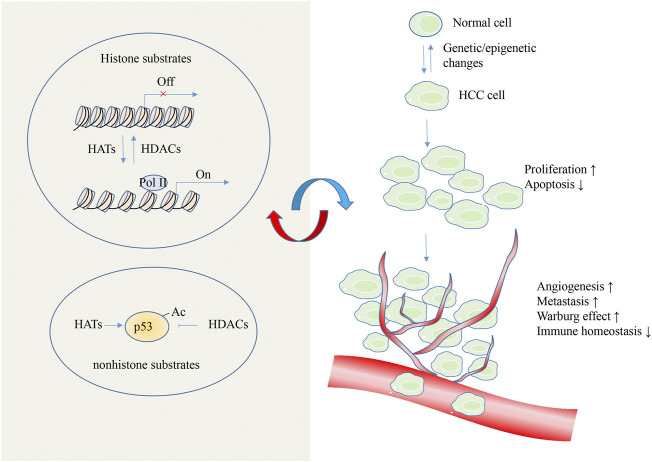
Roles of HATs/HDACs in HCC. Aberrant HATs/HDACs-mediated histone acetylation trigger oncogenes activation, and loss in tumor suppressor gene expression to lead the HCC establishment. p53 was the representative non-histone protein that shown to be acetylated/deacetylated by HATs/HDACs, and this type of modification is essential for p53 activity in HCC. In turn, the metabolic product from HCC also influence the acetylation modifications.

In the last decades, epigenetic modifications has been validated to contribute to the process of various kinds of cancers (Dawson and Kouzarides, 2012). Histone deacetylation is one of the earliest discovered epigenetic mechanisms, regulating many cellular events such as differentiation, proliferation, apoptosis, metabolic changes, metastasis and immune homeostasis in HCC (Figure 2). As a key regulator of acetylation status, HATs/HDACs have been found to dysregulate and/or function incorrectly in HCC, thereby providing a crucial attractive target for HCC treatment (Table 4). Currently, there are numerous HDACIs such as vorinostat, romidepsin, belinostat, panobinostat, tazemetostat, and chidamide are approved by the United States Food and Drug Administration for clinical treatment (Li and Seto, 2016; Ganai, 2020a; Nepali and Liou, 2021). However, opposite regulatory roles of HDACs were observed in HCC. Besides, the efficacies of HDAC inhibitory compounds observed against solid tumours have been disappointing, possibly owing to the lack of specificity. There is reason to believe that maintenance of the balance of histone acetylation modifications is essential for the regulation of gene expression and the maintenance of the normal status of cells. More studies are needed to systematically dissect the role and precise mechanisms of individual HDACs in HCC, which will give United States mechanistic-based rationale for the clinical use of HDACI. In addition, the effectiveness of nonselective HDACI relies on its broad-spectrum inhibition against HDACs, long-term uses of broad spectrum nonselective HDACI are potentially cytotoxic and might induce intolerable side effects in certain patients. Moreover, the activities of HDAC are often mechanistically connected with DNA methylation, miRNAs and lncRNA in HCC (Zhang et al., 2010; Yuan et al., 2011; Ding et al., 2017). Therefore, a combination of epigenetic drugs targeting multiple epigenetic alterations might incur fewer side effects. In parallel, it is anticipated that future research developing HDACI with higher target specificity that might be more efficacious with less toxicity. Because epigenetic modifications occur in a tissue, cell, or gene-specific manner, different targeted genes of HDACI might cause its distinct influences. Thus, further identification of the key genes of acetylation modifications and understanding the underlying regulatory mechanisms might lead to clinical benefits for HCC.

**TABLE 4:** Expression and target genes of HATs/HDACs in HCC.

HATs/HDACs	Differential expression in HCC	Target genes	Biological processes/cellular functions	References
HDAC1	↑	E-cadherin	EMT	Hu et al. (2019)
		CCAAT/enhancer binding protein β (C/EBPβ)	EMT	Huo et al. (2021)
		hypoxia-inducible factor 1α (HIF-1α)	EMT	Yoo et al. (2008); Liu et al. (2013)
		FBP1	gluconeogenesis	Yang et al. (2017)
		P53	Apoptosis	Zhang et al. (2020b)
HDAC2	↑	E-cadherin	EMT	Hu et al. (2019)
		integrin αV subunit gene	cell migration	Cai et al. (2019)
HDAC3	-	E-cadherin	EMT	Hu et al. (2019)
		ANCR	HCC metastasis	Wen et al. (2020)
HDAC6	↓	HIF-1α and VEGFA	angiogenesis	Lv et al. (2016)
HDAC8	↓	PKM2	Glycolysis	Zhang et al. (2020c)
SIRT1	↑	hnRNP A1	Glycolysis	Yang et al. (2019)
		LC3	Autophagy	Li et al. (2016)
		P53	Apoptosis	Lee et al. (2012); Lin et al. (2019)
SIRT2	↑	protein kinase B	EMT	Chen et al. (2013)
SIRT5	↑	cytochrome c	mitochondrial apoptosis	Zhang et al. (2019)
SIRT5	↓	Vimentin	EMT	Guo et al. (2018)
SIRT6	↑	FOXO3a; Beclin-1	EMT	Han et al. (2019)
		hnRNP A1	Glycolysis	Yang et al. (2019)
		Ku70	Apoptosis	Tao et al. (2017)
SIRT7	↑	PGK1	cell proliferation	Hu et al. (2017)
		MEF2D	Immunity	Xiang et al. (2020)
P300	↑	HMGA2	HCC metastasis	Liang et al. (2021)
		DDK1	HCC metastasis	Niu et al. (2020)
		P53	glycogen metabolism	Chen et al. (2019); Di Leo et al. (2019)
		P53	apoptosis	Zhang et al. (2020b)
hMOF	↑	AXL;LGALS1	cell migration	Pote et al. (2020)
PCAF	↓	PGK1	cell proliferation	Hu et al. (2017)
		GLI1	apoptosis	Gai et al. (2015)

## Abbreviations

HCC, hepatocellular carcinoma; HBV, Hepatitis B virus; HCV, hepatitis C virus; NAFLD, non-alcoholic fatty liver disease; HATs, histone acetyltransferases; HDACs, histone deacetylases; Sir2, silent information regulator 2; HBx, hepatitis B virus X protein; EMT, epithelial-mesenchymal transition; E-cadherin; E-cadherin, epithelial-cadherin; α-SMA, α-smooth muscle actin; PKB, protein kinase B; HDACI, histone deacetylase inhibitor; HIFs, hypoxia-inducible factors; PKM2, pyruvate kinase M2 isoform; FBP1, fructose-1,6-bisphosphatase; FASN, fatty acid synthase; EPA, eicosapentaenoic acid; Bax, BCL2-Associated X; HBeAg, hepatitis B e antigen; lncRNA, long non-coding RNA; ICB, immune-checkpoint blockade; MICA and MICB, MHC class I-related chain molecules A and B; MDSCs, myeloid-derived suppressor cells.
